# Impaired ATP6V0A2 expression contributes to Golgi dispersion and glycosylation changes in senescent cells

**DOI:** 10.1038/srep17342

**Published:** 2015-11-27

**Authors:** Miyako Udono, Kaoru Fujii, Gakuro Harada, Yumi Tsuzuki, Keishi Kadooka, Pingbo Zhang, Hiroshi Fujii, Maho Amano, Shin-Ichiro Nishimura, Kosuke Tashiro, Satoru Kuhara, Yoshinori Katakura

**Affiliations:** 1 Graduate School of Bioresources and Bioenvironmental Sciences, Kyushu University, 6-10-1 Hakozaki, Higashi-ku, Fukuoka 812-8581, Japan; 2 Graduate School of Systems Life Sciences, Kyushu University, 6-10-1 Hakozaki, Higashi-ku, Fukuoka 812-8581, Japan; 3 Faculty of Agriculture, Kyushu University, 6-10-1 Hakozaki, Higashi-ku, Fukuoka 812-8581, Japan; 4 Faculty of Advanced Life Science and Graduate School of Life Science, Hokkaido University, N21, W11, Kita-ku, Sapporo 001-0021, Japan

## Abstract

Many genes and signaling pathways have been found to be involved in cellular senescence program. In the present study, we have identified 16 senescence-associated genes by differential proteomic analysis of the normal human diploid fibroblast cell line, TIG-1, and focused on ATP6V0A2. The aim of this study is to clarify the role of ATP6V0A2, the causal gene for ARCL2, a syndrome of abnormal glycosylation and impaired Golgi trafficking, in cellular senescence program. Here we showed that ATP6V0A2 is critical for cellular senescence; impaired expression of ATP6V0A2 disperses the Golgi structure and triggers senescence, suggesting that ATP6V0A2 mediates these processes. FITC-lectin staining and glycoblotting revealed significantly different glycosylation structures in presenescent (young) and senescent (old) TIG-1 cells; reducing ATP6V0A2 expression in young TIG-1 cells yielded structures similar to those in old TIG-1 cells. Our results suggest that senescence-associated impaired expression of ATP6V0A2 triggers changes in Golgi structure and glycosylation in old TIG-1 cells, which demonstrates a role of ATP6V0A2 in cellular senescence program.

Many genes involved in tumor suppression (p53, p21, and p16), p38 MAPK pathway, PI3K/AKT/mTOR pathway, DNA damage response, senescence-associated secretory phenotype (IL-6, IL-8, NF-κB and c/EBPβ) have been associated with cellular senescence^1^,^2^,^3^. Recently, the high-resolution differential proteomic analysis has been used to find proteins that are differentially expressed in senescent cells^4^, however, functionalities of these proteins were not fully understood. We have identified 16 such senescence-associated proteins, of which ATP6V0A2 is the focus of the present study.

ATP6V0A2 is the causal gene in autosomal recessive cutis laxa type 2 (ARCL2), a syndrome of growth and developmental delay, redundant and inelastic skin^5^,^6^. Skin fibroblast derived from ARCL2 patient showed increased apoptosis and further ARCL2 can be regarded as segmental progeroid syndromes showing aging-associated changes in some tissues^5^,^7^, suggesting a link between cellular senescence and the onset of ARCL2 disease.

ATP6V0A2 encodes a subunit of the vacuolar ATPase that acidifies membrane-enclosed organelles including vacuoles, lysosomes, endosomes, coated vesicles and Golgi apparatus. During the transport through the Golgi, proteins are subjected to covalent modifications such as glycosylation. Glycosyltransferases and glycosidases produce variety of glycan structure inside the Golgi. Thus, the genetic defect of ATP6V0A2 is associated with glycosylation abnormalities in Golgi resulting in both *N*- and *O*-glycosylation deficiencies^8^. However, the exact mechanisms for the ATP6V0A2 defect-induced glycosylation abnormality are not fully understood.

The change in glycosylation leads to functional alteration of cells, tissues and organs, and would reflect chronic conditions such as autoimmunity, metabolic syndrome and aging^9^,^10^,^11^. Glycosylation diseases are caused by defects in the machinery in the docking and fusion of transport vesicles, defects in protein sorting apparatus, and defects in Golgi pH homeostasis and membrane fusion. ATP6V0A2 is thought to be a good candidate for probing the cause of human glycosylation diseases.

Considering at a cellular level, the structure of the Golgi complex is altered in senescent cells^12^, which might lead to glycosylation abnormalities^8^. We then assumed that ATP6V0A2 would play a key role in Golgi collapse and subsequent glycosylation abnormalities caused by cellular senescence. By investigating the role of ATP6V0A2 in the maintenance of the Golgi homeostasis and in the cellular senescence, we would clarify the target molecules and mechanisms for treating diseases caused by glycosylation abnormalities.

## Results

Identification of senescence-associated genes by subtractive proteomic analysis.

**Figure 1:**
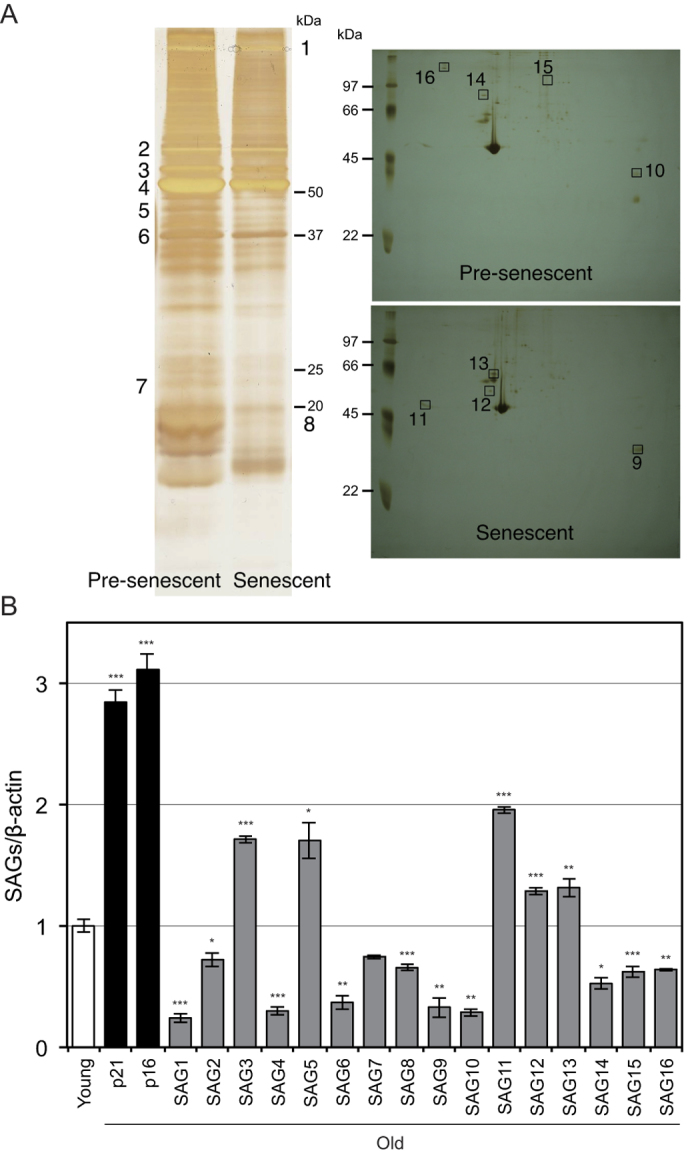
Identification of senescence-associated proteins and their expression profiles. (**A**) Comparison of proteome on Pre-senescent (Young) and Senescent (Old) TIG-1 cells. Proteins from Young TIG-1 cells (37 PDL) and Old TIG-1 cells (61 PDL) were separated by SDS-PAGE (Left) and two-dimensional gel electrophoresis (Right). Identified protein bands and spots are indicated by numbers. (**B**) Relative expression of senescence-associated genes (SAGs) in Old vs. Young TIG-1 cells was determined by qRT-PCR.

We identified 16 novel senescence-associated genes (SAGs) by subtractive proteomic analysis in pre-senescent (young) and senescent (old) human normal diploid cells, TIG-1 (Fig. 1A and Table S1). To validate these results, we analyzed gene expression of these SAGs in young and old TIG-1 cells by quantitative RT-PCR. Senescence marker genes p21 and p16 were upregulated in old TIG-1 cells (Fig. 1B). Although the expression patterns of some SAGs were not coincident with those obtained by proteomic analysis, the expression of ten SAGs were downregulated and those of 5 SAGs were upregulated in old TIG-1 cells. Among these SAGs, we focused on SAG15 for further analysis.

### Functional role of ATP6V0A2 in senescence of TIG-1 cells

**Figure 2:**
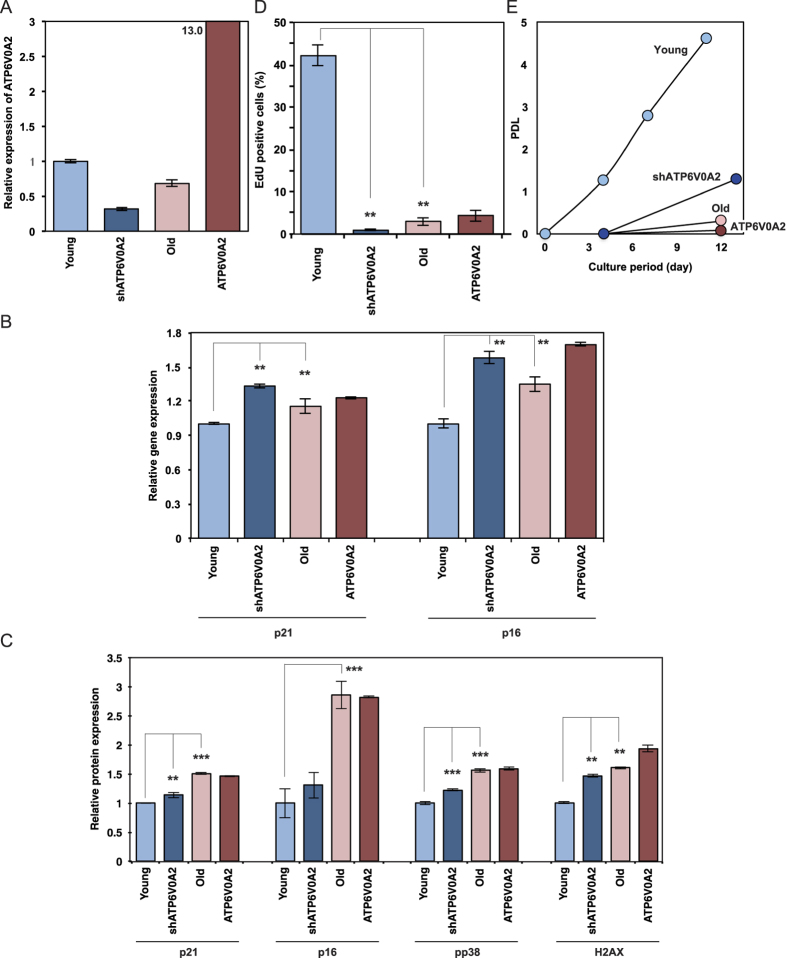
Function of ATP6V0A2 in the cellular senescence program. Relative gene expression of ATP6V0A2 (**A**), p21 and p16 (**B**), relative protein expression of p21, p16, phospho-p38 and γH2AX (**C**), relative EdU positive cells (**D**) and proliferative potential (**E**) in Young TIG-1 transduced with pSUPER.retro (Young; light blue), ATP6V0A2-silenced Young TIG-1 (shATP6V0A2; blue), Old TIG-1 transduced with pBABE-puro (Old; pink), and ATP6V0A2-overexpressing Old TIG-1 (ATP6V0A2; red) were shown.

As shown in Fig. 1B, the expression of SAG15 (ATP6V0A2) is downregulated in old TIG-1 cells, suggesting ATP6V0A2 functions mainly in young TIG-1 cells. Thus, we generated recombinant TIG-1 cells where ATP6V0A2 expression was reduced by shRNA and ATP6V0A2 was overexpressed. As shown in Fig. 2A, ATP6V0A2 expression in old TIG-1 cells and young TIG-1 cells transduced with shATP6V0A2 (ATP6V0A2-silenced young TIG-1 cells) was significantly reduced in comparison to young TIG-1 cells. In contrast, ATP6V0A2 expression in TIG-1 cells overexpressing ATP6V0A2 greatly increased.

**Figure 3:**
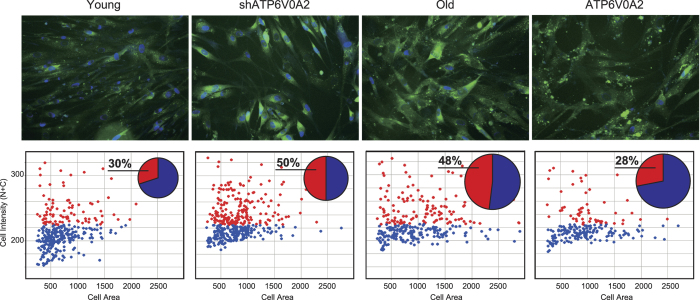
SA-β-Gal assay. Senescence was measured by fluorescence SA-β-Gal assay in Young TIG-1 transduced with pSUPER.retro (Young), ATP6V0A2-silenced Young TIG-1 (shATP6V0A2), Old TIG-1 transduced with pBABE-puro (Old), and ATP6V0A2-overexpressing Old TIG-1 (ATP6V0A2). Nuclei were stained with Hoechst 33342 (Upper). After capturing the cell images, fluorescence intensity and cellular/nuclear size were analyzed with the Developer software and visualized with Spotfire DecisionSite Client 8.2 software. The scatter plot shows whole cell distributions vs. cellular SA-β-Gal activity (Cell Intensity) and cellular size (Cell Area). The pie chart shows the ratio of SA-β-Gal-positive (red)/negative cells (blue). The size of the pie chart represents the average cellular size.

We evaluated the functional role of ATP6V0A2 in cellular senescence of the TIG-1 cells. As shown in Fig. 2B,C, the mRNA expression of senescence markers p21 and p16, and protein expression of senescence markers p21, p16, phospho p38 and γH2AX significantly increased in ATP6V0A2-silenced young and old TIG-1 cells. When DNA replication was assayed by examining 5-ethynyl-2′-deoxyuridine (EdU) incorporation in a pulse-label experiment, there was a steady decrease in EdU incorporation in ATP6V0A2-silenced young TIG-1 cells and old TIG-1 cells (Fig. 2D). Growth was greatly reduced in ATP6V0A2-silenced young TIG-1 cells and old TIG-1 cells (Fig. 2E). The activity of senescence-associated β-galactosidase (SA-β-Gal) was augmented in ATP6V0A2-silenced young TIG-1 cells and old TIG-1 cells (Fig. 3). Furthermore, senescence induction by reduced expression of ATP6V0A2 was also observed in another human normal fibroblast, HUC-F2 (data not shown). These results demonstrate reduced expression of ATP6V0A2 is not the result of senescence, but triggers senescence in TIG-1 cells.

In addition, rescue experiments were done by overexpressing ATP6V0A2 in old TIG-1 cells. Although the growth phenotype and activity of relating molecules in old TIG-cells were not resumed, SA-β-Gal activity in old TIG-1 cells were suppressed by overexpressing ATP6V0A2. These results suggest that old TIG-1 cells partially reverses the signs of cellular senescence by ATP6V0A2.

### Changes in Golgi structure

The Golgi apparatus is dispersed in senescent cells^12^ and disrupted from a ribbon structure into mini-stacks by reducing the expression of ATP6V0A2 in HeLa cells^5^. We then evaluated their similarities by using young TIG-1 cells, ATP6V0A2-silenced young TIG-1 cells, old TIG-1 cells, and ATP6V0A2-overexpressing old TIG-1 cells. The Golgi structure was examined by immunofluorescence microscopy using antibodies specific for GM130, which localizes to the cis Golgi, and TGN46, which is the endogenous trans Golgi network marker. Golgi structure disruption was quantified by counting the cells with dispersed Golgi.

**Figure 4:**
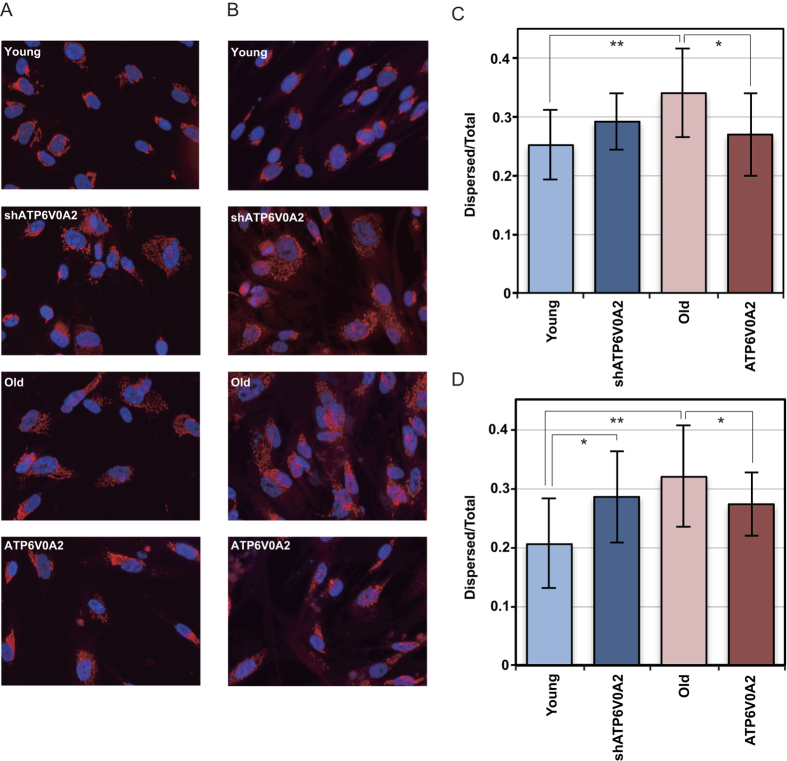
Golgi structure in TIG-1 cells. Golgi structures in YoungTIG-1 cells, ATP6V0A2-silenced Young TIG-1 cells, Old TIG-1 cells, and ATP6V0A2-overexpressing Old TIG-1 cells were evaluated by immunofluorescence with anti-GM130 antibody (**A**) and anti-TGN46 antibody (**B**). Nuclei were counterstained by Hoechst 33342. The number of cells in which distance between nucleus and fluorescence signals of GM130 and TGN46 is longer than the threshold level (cells with dispersed Golgi) were counted by the Developer software and the ratio of cells with dispersed Golgi vs. total cells is shown in (**C**) and (**D**). Data were analyzed by two-sided Student’s t-test. Differences at p < 0.05 were considered significant (*p < 0.05; **p < 0.01).

The cis Golgi structure detected by GM130 was compact in young TIG-1 cells and dispersed in old TIG-1 cells. Although the cis Golgi structure of young TIG-1 cells tended to disperse upon reducing the expression of ATP6V0A2, this change was not significant (Fig. 4A,C). In contrast, the trans Golgi structure detected by TGN46 was significantly dispersed in old and ATP6V0A2-silenced young TIG-1 cells versus young TIG-1 cells. Furthermore, the dispersed Golgi structure in old TIG-1 cells was significantly recovered by ATP6V0A2. These results suggest the Golgi structure was dispersed in young TIG-1 cells when ATP6V0A2 is suppressed and compacted in old TIG-1 cells when ATP6V0A2 is expressed; disruption of the Golgi structure in old TIG-1 cells might be triggered by the reduction of ATP6V0A2 expression, and recovered by ATP6V0A2 expression.

On the other hand, IL-8 secretion was significantly altered in ATP6V0A2-silenced young TIG-1 cells and old TIG-1 cells, as compared to young TIG-1 cells, suggesting that disruption of Golgi structure led to disturbance in the secretion of IL-8 (Fig. S1).

### Changes in glycosylation

**Figure 5:**
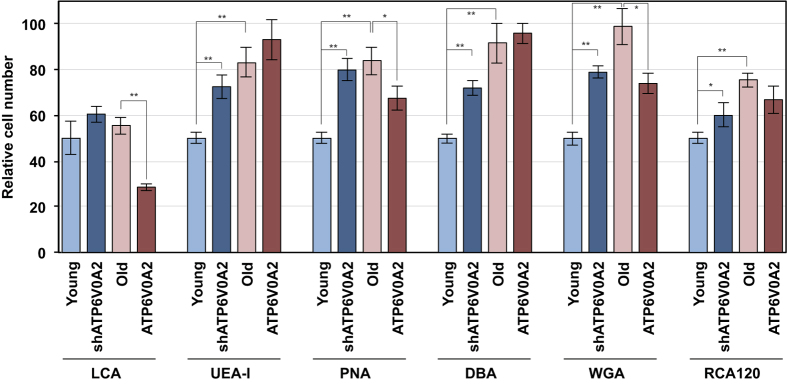
Cellular glycosylation status. Young, ATP6V0A2-silenced Young TIG-1 cells, Old TIG-1 cells and ATP6V0A2-overexpressing Old TIG-1 cells were stained with FITC-lectins. After capturing cell images with IN Cell Analyzer 1000, fluorescence intensity and cell number were analyzed with Multi Target Analysis software. The relative number of cells having fluorescence intensity more than mean fluorescence intensity in Young TIG-1 cells was shown.

The loss-of-function mutations in ATP6V0A2 detected in families with ARCL2 or wrinkle skin syndrome result in abnormal glycosylation of serum proteins^6^. We attempted to detect changes in glycosylation in ATP6V0A2-silenced young TIG-1 cells, old TIG-1 cells, and ATP6V0A2-overexpressing TIG-1 cells versus young TIG-1 cells by using FITC-labeled lectins. We used six lectins—LCA specific for α-D-mannose and α-D-glucose, UEA-I for α-L-fucose, PNA for β-Gal(1,3)GalNAc, DBA for α-GalNAc, WGA for GlcNAc, and RCA120 for galactose. Relative FITC intensity per cell was measured by IN Cell Analyzer 1000 and the relative number of cells having fluorescence intensity more than mean fluorescence intensity of young TIG-1 cells was shown in Fig. 5. Relative FITC intensities increased in old versus young TIG-1 cells, suggesting glycosylation binding by each lectin increased in old TIG-1 cells. Furthermore, relative FITC intensities in ATP6V0A2-silenced young TIG-1 cells were similar to those in old TIG-1 cells, indicating that reducing ATP6V0A2 expression shifted the glycosylation pattern in young TIG-1 cells toward that found in old TIG-1 cells. Conversely, ATP6V0A2 expression shifted some of the glycosylation pattern in old TIG-1 cells toward that found in young TIG-1 cells.

**Figure 6:**
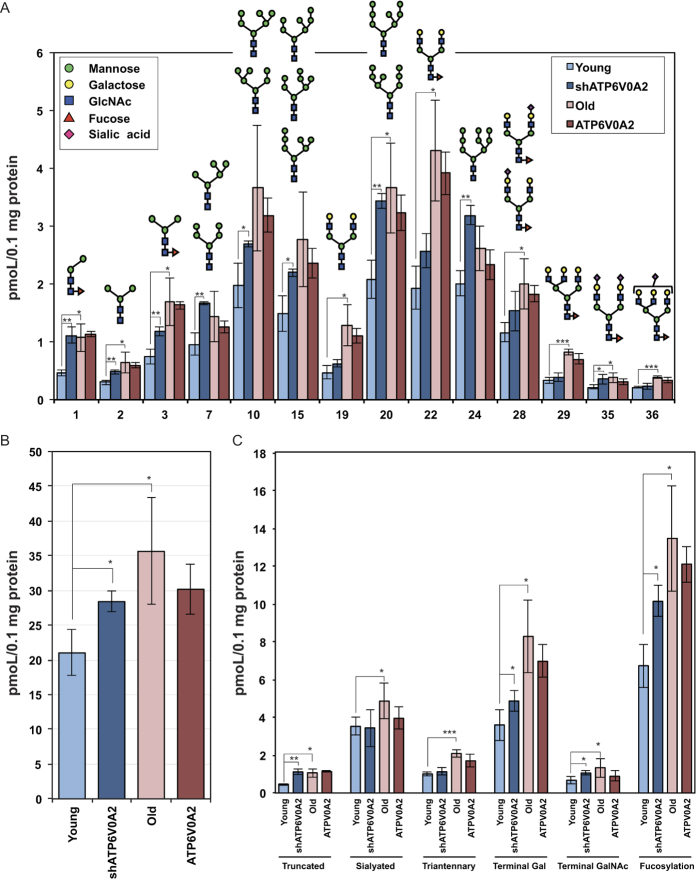
Quantitative analysis of *N*-glycans during cellular senescence. Quantitative *N*-glycan profiling in Young TIG-1 cells, ATP6V0A2-silenced Young TIG-1 cells, Old TIG-1 cells, and ATP6V0A2-overexpressing Old TIG-1 cells are shown. (**A**) Of 41 identified *N*-glycans, 14 glycoforms (peak numbers 1, 2, 3, 7, 10, 15, 19, 20, 22, 24, 28, 29, 35, 36) were shown. (**B**) Amount of total *N*-glycans in Young TIG-1 cells, ATP6V0A2-silenced TIG-1 cells, old TIG-1 cells, and ATP6V0A2-overexpressing TIG-1 cells are shown. (**C**) Comparison of glycan features. Glycan features represent summed data of individual glycan.

To characterize the changes in TIG-1 cell glycosylation patterns, we performed glycoblotting analysis. Total *N*-glycans collected from young TIG-1 cells, ATP6V0A2-silenced young TIG-1 cells, old TIG-1 cells, and ATP6V0A2-overexpressing TIG-1 cells were analyzed and identified by a combination of glycoblotting and MALDI-TOF MS^13^,^14^. Total *N*-glycan levels increased in old TIG-1 cells (Fig. 6B). This increase was also detected in ATP6V0A2-silenced young TIG-1 cells. Furthermore, 41 different *N*-glycans were detected and quantified in these cells. Among these glycans, nine (Peak No. 1, 2, 3, 19, 20, 22, 29, 35, and 36) increased significantly in old TIG-1 cells (Fig. 6A). On the other hand, eight glycans (Peak No. 1, 2, 3, 7, 10, 15, 20, and 35) increased significantly in ATP6V0A2-silenced young TIG-1 cells. These results suggest glycosylation patterns significantly differ between young and old TIG-1 cells, and that the patterns in ATP6V0A2-silenced young TIG-1 cells were similar to those in old TIG-1 cells. In addition, ATP6V0A2 expression tended to shift some glycans in old TIG-1 cells toward that found in young TIG-1 cells.

The quantitative analysis of glycan features was shown in Fig. 6C. All glycan features increased in old TIG-1 cells. Among them, truncated glycan, terminal Gal glycan, terminal GalNAc glycan and fucosylated glycan also increased in ATP6V0A2-silenced young TIG-1 cells. ATP6V0A2 expression only slightly decreased some of glycan features in old TIG-1 cell. All these results suggest that glycan features in ATP6V0A2-silenced young TIG-1 cells are also similar to those in old TIG-1 cells.

## Discussion

Critical pathways and genes associated with cellular senescence have been identified by varied approaches^15^,^16^. In this study, we identified 16 senescence-associated genes by subtractive proteomics in young and old TIG-1 cells (Table S1), some of which have been pointed out about their relationship with senescence^17^. However, most of these genes have not been reported as senescence-associated genes. Interaction network of these genes was analyzed by in silico analysis (Ingenuity Pathway Analysis (IPA)) (Fig. S2), suggesting an involvement of ubiquitination-related mechanisms in cellular senescence. Among these genes, overexpression of SAG5 and SAG11, and knockdown of SAG1, SAG8, SAG10 and SAG15 induced cellular senescence. Knockdown of SAG15 (ATP6V0A2) showed most strong senescence-inducing ability, then we focused on ATP6V0A2 and found by *in vitro* functional analysis that it functions as an anti-senescence gene.

ATP6V0A2 is a subunit of the multimeric vacuolar H^+^-ATPase (v-ATPase) enzyme transporter. Kurz *et al.* reported that lysosomal alkalinisation by the treatment with a specific inhibitor of v-ATPase, bafilomysin A1, did not induce cellular senescence^18^. This result suggests that ATP6V0A2 induces cellular senescence not only by the functional depletion of the v-ATPase, but also through the intermediary of other mechanisms.

Mutations in the ATP6V0A2 gene result in abnormal glycosylation of serum proteins and impair Golgi trafficking in the fibroblasts of affected individuals^6^, and reduced expression of ATP6V0A2 leads to disruption of the Golgi structure^5^. Furthermore, the Golgi structure is dispersed in senescent cells^12^. Thus, these results suggest ATP6V0A2 contributes to the Golgi structure disruption and corresponding changes in glycosylation in senescent cells. Indeed, we detected disruption of the Golgi structure in old TIG-1 cells with reduced ATP6V0A2 expression and significant differences in glycosylation between young and old TIG-1 cells, and observed glycosylation patterns in young TIG-1 cells with reduced ATP6V0A2 expression similar to those in old TIG-1 cells (Fig. 6). These results suggest the disruption of Golgi structure and the altered glycosylation pattern in old TIG-1 cells is caused by the senescence-induced impairment of ATP6V0A2 expression. Furthermore, inhibition of the clathrin-mediated trafficking at the plasma membrane and the TGN has been reported to induce senescence by inducing lysosomal instability and iron leakage^19^, which suggests an involvement of similar mechanisms.

The precise mechanism by which mutations in the ATPV0A2 subunit affect Golgi structure and glycosylation patterns has been unclear. ATP6V0A2 is known to play an important role in medial- and trans-Golgi pH acidification and in retrograde membrane trafficking^20^. This lumeneal pH regulation is crucial for posttranslational modification in the Golgi compartment^21^. Altered function or reduced expression of ATP6V0A2 disturbs the Golgi pH, which affects the activity and localization of certain Golgi glycosyltransferases and/or glycosylation due to a lack of fusion of vesicles containing Golgi glycosyltransferases^8^, which results in the glycosylation change. Thus, the Golgi apparatus and glycosylation pattern would be affected by senescence-associated impairment of ATP6V0A2 expression. This impaired Golgi trafficking and glycosylation would trigger Golgi stress and further cellular senescence. In addition, as a result of Golgi dispersion, changes in production and glycosylation of secretory proteins would form positive feedback loop and contribute to induce or enhance cellular senescence phenotypes.

Glycoblotting analysis revealed increases in sialylated and fucosylated sugar chains (Fig. 6A, Peak No. 35 and 36, Fig. 6C) and fucosylated lactosamines (Fig. 6A, Peak No. 22 and 29) in old TIG-1 cells. Furthermore, glycan features including sialyated glycan, terminal Gal glycan and fucosylated gycan increased in old TIG-1 cells (Fig. 6C). The increase in sialylated and fucosylated sugar chains has also been observed in the serum of patients with inflammatory diseases and mice during inflammation^22^,^23^, suggesting this sugar chain structure might reflect cellular inflammatory status. An increase in fucosylated lactosamine has been also detected in cells stably transfected with senescence-associated factor p16INK4a^24^, suggesting this sugar chain structure might be a marker of cellular senescence.

Most of these changes in glycosylation pattern observed in old TIG-cells were detected in ATP6V0A2-silenced TIG-1 cells. These results indicate that similar events, such as reduced expression of ATP6V0A2, dispersion of Golgi structure, and corresponding change in glycosylation, occur both in old TIG-1 cells and ATP6V0A2-silenced cells, which suggests that ATP6V0A2 plays a key role in maintaining normal glycosylation, and that cellular senescence-induced repression of ATP6V0A2 contributes to Golgi dispersion and glycosylation changes in senescent cells. All these results suggest that ATP6V0A2 might be a potential target for suppressing senescence and further treating diseases caused by glycosylation abnormalities.

## Methods

### Cell culture

TIG-1 cells (Cell Resource Center for Biomedical Research, Tohoku University, Miyagi, Japan) were cultured in MEM (Nissui, Tokyo, Japan) supplemented with 10% fetal bovine serum (FBS; HyClone, HyClone, Logan, Utah); HEK293 cells (JCRB9068; HSRRB, Osaka, Japan) in DMEM (Nissui) supplemented with 10% FBS at 37 °C in 5% CO_2_.

### Identification of senescence-associated proteins

Proteins from young TIG-1 cells (37 population doubling levels (PDL)) and old TIG-1 cells (61 PDL) were separated by SDS-PAGE and two-dimensional gel electrophoresis. Protein bands and spots exhibiting differential expression were excised and subjected to in-gel digestion (Thermo Fisher Scientific, Waltham, MA). Peptide masses were analyzed by MALDI-TOF MS using a Voyager-DE RP Biospectrometry workstation (Applied Biosystems, Framingham, MA). Peptide masses were searched against Swiss-Prot and NCBI databases using the Aldente search tool, as described previously^25^.

### Retrovius production and transduction

Viral supernatants were produced after transduction of 293T cells with pGag-pol, pVSV-G, and individual expression vector (pBABE-puro-ATP6V0A2 or mock) using the HilyMax reagent (Dojin, Kumamoto, Japan) as previously described^26^. The target cells were infected with this viral supernatant for 24 h at 37 °C. After infection, the cells were selected with 3 μg/mL puromycin (Enzo Life Sciences, Farmingdale, NY) for 3 days.

### Short hairpin RNA (shRNA)

Oligonucleotides containing the siRNA-expressing sequence targeting ATP6V0A2 were annealed (shATPV0A2 top: 5′-GATCCCCGCAGCTTTGACGTGACCAACATTCGAAGAGTGTTGGTCACGTCAAAGCTGCTTTTTA-3′, shATP6V0A2 bottom: 5′-AGCTTAAAAAGCAGCTTTGACGTGACCAACACTCTTCGAATGTTGGTCACGTCAAAGCTGCGGG-3′), and cloned into the pSUPER.retro vector (OligoEngine, Seattle, WA). Preparation of viral supernatant and infection of target cells was performed as described^26^. Transduced cells were selected with 2 μg/mL puromycin for 3 days.

### Quantitative RT-PCR (qRT-PCR)

RNA was isolated using the High Pure RNA Isolation Kit (Roche, Mannheim, Germany). cDNA was synthesized using the ReverTra Ace Kit (Toyobo, Osaka, Japan). qRT-PCR was performed using the KAPA SYBR FAST qPCR Kit (KAPA Biosystems, Woburn, MA) and the Thermal Cycler Dice Real Time System TP-800 instrument (Takara, Shiga, Japan), as described^27^. The samples were analyzed in triplicate, and gene expression levels were normalized to β-actin. The PCR primers are listed in Table S2.

### Fluorescence SA-β-Gal assay

The fluorescence SA-β-Gal assay was performed by using fluorescent substrate of β-galactosidase (ImaGene Green C_12_FDG; Life Technologies, Gaithersburg, MD) as previously described^28^. The image of each well was acquired by using IN Cell Analyzer 1000 (GE Healthcare, Amersham Place, UK). Hoechst 33342 (Dojin) stain was used to generate cell counts. Imaging data were reported as SA-β-Gal activity (mean fluorescence intensity per cell) and the SA-β-Gal positive/negative ratio was determined by setting the threshold intensity of the SA-β-Gal staining at which ~75% of control cells were negative for SA-β-Gal activity. Data collected using Developer were imported into Spotfire DecisionSite Client 8.2 software to visualize the results.

### DNA synthesis assay

DNA synthesis was assayed with EdU method using the Click-iT Plus EdU Alexa Fluor 555 imaging reagent (ThermoFisher Scientific). The stained cells were counted using IN Cell Analyzer 1000.

### Immunofluorescence

Cell fixation and blocking were performed according to manufacturer instructions. Cells were incubated with antibodies (anti-p21, Cell Signaling Technology (Danvers, MA); anti-p16, Abcam (Cambridge, MA); anti-phospho-p38 MAPK (Thr180/Tyr182), Cell Signaling Technology; anti-γH2AX, Cell Signaling Technology; anti-GM130, BD Bioscience, Clontech, Palo Alto, CA; anti-TGN46, Sigma, St Louis, MO) and with Alexa Fluor 555-labeled secondary antibody F(ab’) fragment (Life Technologies). After washing the cells with PBS, cells were incubated with 1 μg/mL Hoechst 33342 solution for 30 min. Each well was imaged by using IN Cell Analyzer 1000, analyzed by Developer, and visualized by Spotfire DecisionSite Client 8.2 as described above.

### Enzyme-linked Immunosorbent Assay (ELISA)

Human CXCL8/IL-8 Quantikine HS ELISA kit (R&D Systems, Minneapolis, MN) was used to determine the concentration of IL-8 in 72-h-conditioned cell culture supernatant according to the instruction from the manufacturer.

### Evaluation of glycosylation status by lectin binding

Cells were seeded on μClear Fluorescence Black Plates (Greiner bio-one, Tokyo, Japan) and fixed with 2.5% glutaraldehyde. After washing, the cells were incubated with 0.1% TritonX-100 and blocked with 1% BSA. After washing, Hoechst 33342 solution was added. Data collection and analysis were performed as described above.

### Extraction of *N*-glycans from cells and cell culture supernatants

Total cellular *N*-glycans were released as described^13^,^24^ with minor modifications. Trypsinized cells were lysed by 10% of Triton X-100 for 50 min on ice and sonicated at room temperature for 10 min. The lysates were centrifuged and the supernatants incubated with cold acetone (final concentration 80%) overnight at –20 °C to precipitate the protein fraction. After washing, the cell precipitates were dissolved with 0.02% of 1-propanesulfonic acid and 2-hydroxyl-3-myristamido (PHM) and incubated at 60 °C for 10 min. Aliquots (20 μL) of the cell culture supernatants were also mixed with the same PHM-containing buffer. The samples were reduced by incubation in 10 mM 1,4-dithiothreitol (DTT) at 60 °C for 30 min, and then alkylated by incubation with 20 mM iodoacetamide in the dark at room temperature for 30 min. The mixture was incubated with 80 U trypsin at 37 °C overnight, followed by heat-inactivation. After cooling to room temperature, the *N*-glycans were released from the trypsin-digested glycopeptides by overnight incubation at 37 °C with 2 U Peptide-*N*-glycosidase F (PNGase F, Roche). Samples were then dried in a SpeedVac and stored at –20 °C until use. Protein concentrations were determined for all lysates and supernatants by BCA protein assay kit (Thermo Fisher Scientific) according to manufacturer instructions.

### Glycoblotting

*N*-glycans were analyzed as described^13^,^14^. In short, a 250-μL aliquot of BlotGlyco H beads (Sumitomo Bakelite Co., Tokyo, Japan) was reacted with 20 μL PNGase F-digested sample, adjusted to 100 μg protein/20 μL water, with 180 μL of 2% acetic acid in acetonitrile (ACN) at 80 °C for 45 min so that the total glycans were specifically captured by the beads via stable hydrazone bonds. The beads were serially washed and unreacted hydrazide functional groups on the beads were capped by incubation with 10% acetic anhydride in MeOH for 30 min at room temperature. After serial washing, on-bead methyl esterification of the carboxyl groups of sialic acids was carried out by incubation with 150 mM 3-methyl-1-ptolyltriazene in dioxane at 60 °C until dryness. The beads were then serially washed and subjected to transiminization by incubation with aminooxy-functionalized tryptophanyl arginine (ao-WR) for 45 min at 80 °C. The ao-WR-tagged glycans were released by adding 100 μL of water, and then purified using a Mass PREP™ HILIC μElution Plate (Waters, Milford, MA) according to manufacturer instructions.

### MALDI-TOF/MS and data analysis

Purified *N*-glycans were concentrated 10-fold in a SpeedVac and then mixed with 2,5-dihydroxylbenzoic acid (10 mg/mL in 30% ACN) (1:2) and allowed to crystallize. Analytes were subjected to matrix-assisted laser desorption ionization-time of flight (MALDI-TOF) mass spectrometry, Ultraflex III (BrukerDaltonics, Billerica, MA), operated in reflector, positive ion mode, typically summing 7,000–10,000 shots until the peak intensity of the internal standard reached 1 × 10^5^. *N*-glycan spectral peaks were picked using FlexAnalysis ver. 3.0 software (Bruker Daltonics) and in-house software. Quantitation was performed by normalizing the area of the corresponding peak of each glycan to that obtained with 20 pmol of an internal standard (A2 amide glycan) which was mixed into the samples just before glycoblotting. Glycan structures were predicted using the GlycoMod Tool (http://web.expasy.org/ glycomod/) and Glycosuite DB (http://glycosuitedb.expasy.org/glycosuite/glycodb). Glycotype analysis was performed as described^14^, based on the predicted glycan structures.

### Statistics

All experiments were performed at least three times, and the corresponding data are shown. The results are expressed as mean ± standard error of the mean. Statistical significance was determined using a two-sided Student’s t-test. Statistical significance was defined as P < 0.05 (*P < 0.05; **P < 0.02; ***P < 0.001).

## Additional Information

**How to cite this article**: Udono, M. *et al.* Impaired ATP6V0A2 expression contributes to Golgi dispersion and glycosylation changes in senescent cells. *Sci. Rep.* **5**, 17342; doi: 10.1038/srep17342 (2015).

## Supplementary Material

Supplementary Information
