# Supplementary material for: Impaired ATP6V0A2 expression contributes to Golgi dispersion and glycosylation changes in senescent cells
Source: Sci Rep. 2015 Nov 27;5:17342. doi: 10.1038/srep17342 (PMC4661525; doi:10.1038/srep17342)
Supplement: Supplementary Information [file srep17342-s1.pdf]

Supplementary information

Impaired ATP6V0A2 expression contributes to Golgi dispersion and glycosylation changes in senescent cells

Miyako Udono, Kaoru Fujii, Gakuro Harada, Yumi Tsuzuki, Keishi Kadooka, Pingbo Zhang, Hiroshi Fujii, Maho Amano, Shin-Ichiro Nishimura, Kosuke Tashiro, Satoru Kuhara, Yoshinori Katakura

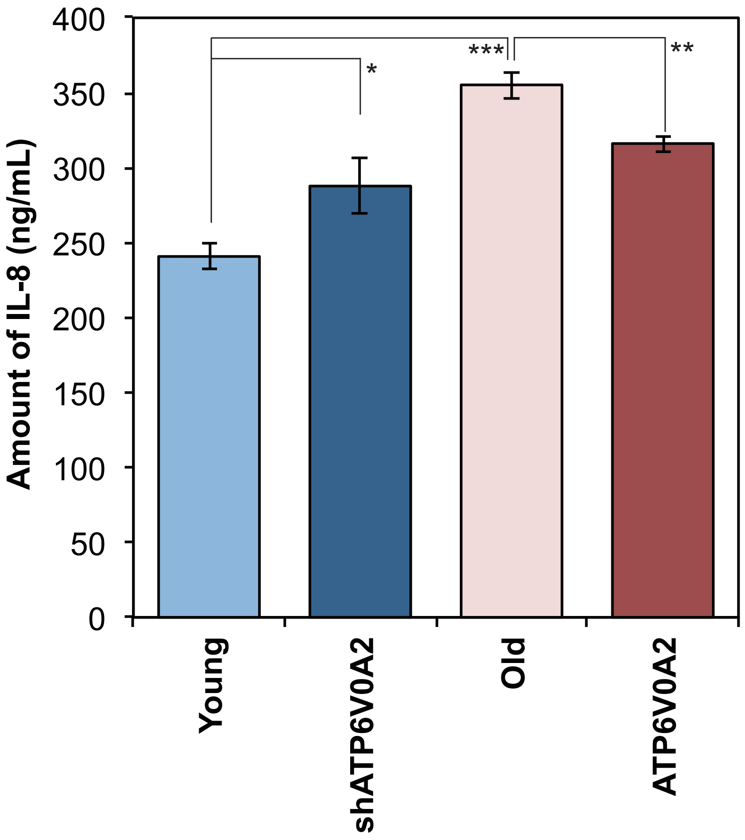

Fig. S1 Effects of ATP6V0A2 on the secretion of IL-8. The amount of IL-8 secreted into the supernatant of Young TIG-1, ATP6V0A2-silenced Young TIG-1, Old TIG-1, and ATP6V0A2-overexpressing TIG-1 were determined by ELISA.

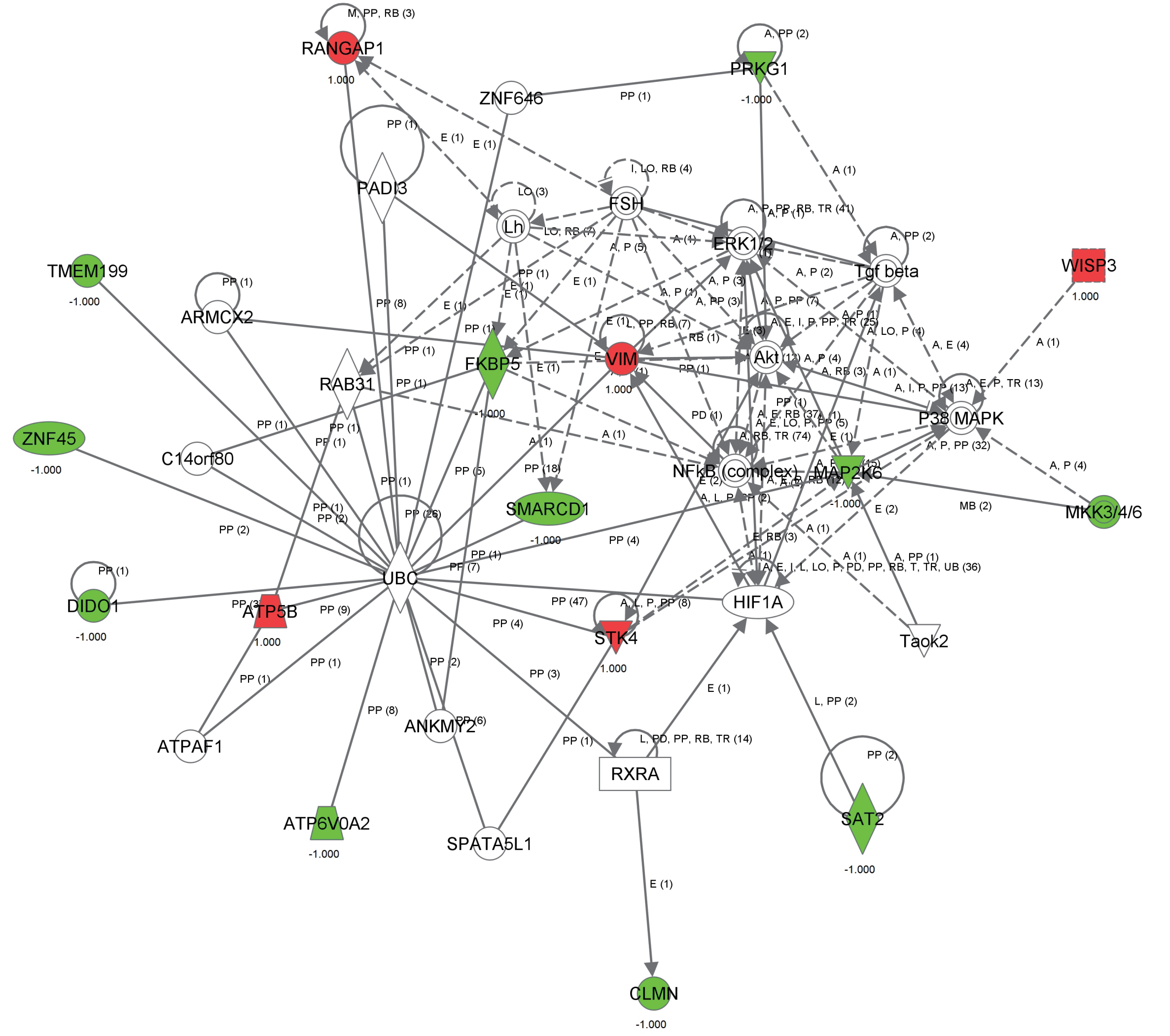

Fig. S2 Interaction network analysis of senescence-associated genes based on information from the Ingenuity Pathway Knowledge Base. Genes that are up- and down-regulated in senescent cells are in red and green, respectively.

Table S1 Senescence-associated genes (SAG)

ID	Gene name
SAG1	Homo sapiens death inducer-obliterator 1 (DIDO1)
SAG2	Home sapiens zinc finger protein 45 (ZNF45)
SAG3	Homo sapiens Ran GTPase activating protein 1 (RANGAP1)
SAG4	Homo sapiens FK506 binding protein 5 (FKBP5)
SAG5	Homo sapiens WNT1 inducible signaling pathway protein 3 (WISP3)
SAG6	Homo sapiens mitogen-activated protein kinase kinase 6 (MAP2K6)
SAG7	Homo sapiens transmembrane protein 199 (TMEM199)
SAG8	Homo sapiens spermidine/spermine N1-acetyltransferase family member 2 (SAT2)
SAG9	Homo sapiens zinc finger protein 73 (ZNF73)
SAG10	Homo sapiens SWI/SNF related, matrix associated, actin dependent regulator of chromatin, subfamily d, member 1 (SMARCD1)
SAG11	Homo sapiens serine/threonine kinase 4 (STK4)
SAG12	Homo sapiens ATP synthase, H+ transporting, mitochondrial F1 complex, beta polypeptide (ATP5B)
SAG13	Homo sapiens vimentin (VIM)
SAG14	Homo sapiens protein kinase, cGMP-dependent, type I (PRKG1)
SAG15	Homo sapiens ATPase, H+ transporting, lysosomal V0 subunit a2 (ATP6V0A2)
SAG16	Homo sapiens calmin (calponin-like, transmembrane) (CLMN)

Table S2 Primers for qRT-PCR

Gene	Primer sequence
Human β-actin	5'-TGGCACCCAGCACAATGAA-3'
	5'-CTAAGTCATAGTCCGCCTAGAAGCA-3'
Human p16	5'-GGCACCAGAGGCAGTAACCA-3'
	5'-GGACCTTGGGTGACTGATGATC-3'
Human p21	5'-TGAAATCGTCCAGCGACCTTC-3'
	5'-GTCCATAGCCTCTACTGCCACC-3'
SAG1 (DIDO1)	5'-GCCTGTCTCCCTGGAGGATT-3'
	5'-TTTCAGAAGAGGCTGGTCGT-3'
SAG2 (ZNF45)	5'-GACGTGGCTGTGGTCTTCTC-3'
	5'-CTCTGGGTTGCCATCTTCAT-3'
SAG3 (RANGAP1)	5'-CTCAGTGATGACGAGGACGA-3'
	5'-CCCAGTGTTAGGGTCCAGAA-3'
SAG4 (FKBP5)	5'-TCCCTCGAATGCAACTCTCT-3'
	5'-AAACATCCTTCCACCACAGC-3'
SAG5 (WISP3)	5'-CAGATGCACCTCAGCGTAAA-3'
	5'-ATCCACAGCCATTCTTCACC-3'
SAG6 (MKK6)	5'-ACGGCTACTGATGGATTTGG-3'
	5'-TGACCGAGAGCATTGATGAG-3'
SAG7 (TMEM199)	5'-CTCCACGGAACCCAGAACTA-3'
	5'-GATGGTGATGACCAGAGCCT-3'
SAG8 (SAT2)	5'-AGTACATGGAAGGGACGCAC-3'
	5'-CTTTCCTGCCAACTTTCTCG-3'
SAG9 (ZNF186)	5'-GGGAAAACGTTTCACCAAAAG-3'
	5'-CATGCATATGGTTTTTCCCCCG-3'
SAG10 (SMARCD1)	5'-ACGTCCCATCAAGCAAAAAC-3'
	5'-TTGGACAAGGCTGAATCCTC-3'
SAG11 (STK4)	5'-TCCTGTGGAATCAGACCTCC-3'
	5'-TCAGATACAGAACCAGCCCC-3'
SAG12 (ATP5B)	5'-TCACCCAGGCTGGTTCAGA-3'
	5'-AGTGGCCAGGGTAGGCTGAT-3'
SAG13 (Vimentin)	5'-GAGAACTTTGCCGTTGAAGC-3'
	5'-GAAATCCTGCTCTCCTCGC-3'
SAG14 (PRKG1)	5'-CGAGGTTATGCCAAACTGGT-3'
	5'-GGATGATCTCTGGGGCTACA-3'
SAG15 (ATP6V0A2)	5'-GGGATTTGTGTCTGGCCTAA-3'
	5'-CAGGGTCTTCAAGGGATTCA-3'
SAG16 (CLMN)	5'-TGTTCAGTTGAGGAACGCAG-3'
	5'-CAAGCTGTGTCAGGGAGTCA-3'
